# Physical Mapping of a Powdery Mildew Resistance Gene in Chromosome 6St from Wheat-*Thinopyrum intermedium* Introgression Lines

**DOI:** 10.3390/plants15091308

**Published:** 2026-04-24

**Authors:** Chengzhi Jiang, Min Wan, Tingting Jiang, Jessy Yee Ting Tan, Aly Boro, Ennian Yang, Zujun Yang, Guangrong Li

**Affiliations:** 1 School of Life Science and Technology, University of Electronic Science and Technology of China, Chengdu 610054, China; 202311140626@std.uestc.edu.cn (C.J.); 202511140604@std.uestc.edu.cn (M.W.); 202421140401@std.uestc.edu.cn (T.J.); 202424140101@std.uestc.edu.cn (J.Y.T.T.); 202514140102@std.uestc.edu.cn (A.B.); 2 Crop Research Institute, Sichuan Academy of Agricultural Sciences, Chengdu 610066, China; yangennian@126.com

**Keywords:** *Thinopyrum intermedium*, powdery mildew resistance, ND-FISH

## Abstract

Powdery mildew, caused by *Blumeria graminis* f. sp. *tritici*, is a devastating disease threatening global wheat production. *Thinopyrum intermedium*, a wild relative of wheat, harbors valuable resistance genes for wheat improvement. In this study, we characterized a wheat-*Th. intermedium* double disomic substitution line X482 (4St-J^S^ (4D) + 6St (6D)) and wheat-*Th. intermedium* partial amphiploid TA8034 using ND-FISH and Oligo-FISH painting. Importantly, a unique type of translocation between chromosomes 1B and 6B was identified in line X482. Subsequent immunostaining assays revealed the distinct DNA methylation maintained on *Thinopyrum* and wheat B-genome chromosomes in X482 and TA8034. Genetic analysis combined with ND-FISH of line X482-derived populations demonstrated that chromosome 6St confers adult-stage powdery mildew resistance. Through ^60^Co-γ irradiation of a 6St monosomic addition line, we developed seven homozygous 6St translocation lines with various breakpoints. Physical mapping using 56 6St-specific molecular markers delineated the resistance locus to bin 6StL-3, corresponding to 358.03–398.21 Mb in the *Th. intermedium* reference genome v2.1. Notably, the wheat-6St translocation line exhibited powdery mildew resistance without significant negative effects on agronomic traits. These results indicated that the distal region of homologous group 6 chromosomes harbors novel powdery mildew resistance genes, and the developed translocation lines provide valuable germplasm for wheat breeding programs.

## 1. Introduction

Common wheat (*Triticum aestivum* L.) is a staple cereal cultivated worldwide, serving as a primary source of nutrition for over one-third of the global population [1,2]. Powdery mildew, caused by the biotrophic fungal pathogen *Blumeria graminis* f. sp. *tritici* (*Bgt*), is a devastating wheat disease that leads to substantial yield losses in major wheat-producing regions globally [3,4]. Conventional control strategies, such as fungicide application, face limitations due to environmental concerns and rising production costs. Developing resistant cultivars is considered the most economical and effective method to manage this disease. To date, seventy-one *Pm* genes (*Pm1*-*Pm71*) have been formally cataloged and more than 100 quantitative trait loci (QTLs) have been documented [5,6]. However, due to pathogen–host coevolution and the rapid evolution of *Bgt* pathotypes, most commercially deployed powdery mildew resistance genes have progressively lost efficacy [7]. Consequently, there is a critical imperative to discover, characterize, and deploy novel powdery mildew resistance loci from wheat-relative germplasm to overcome this vulnerability and advance the breeding of resistant wheat varieties.

*Thinopyrum intermedium* (Host) Barkworth and D.R.Dewey (JJJ^S^J^S^StSt, 2n = 6x = 42) is a perennial wild relative within the Triticeae tribe and possesses numerous excellent characteristics, such as tolerance to abiotic stresses (drought, salinity, and alkalinity) [8,9,10], and broad-spectrum resistance to multiple wheat diseases, including stem rust (*Sr44*), stripe rust (*Yr50*), leaf rust (*Lr38*), and powdery mildew (*Pm40* and *Pm43*) [11,12,13,14,15]. To make these favorable genes from *Th. intermedium* accessible for wheat breeding, extensive hybridizations between wheat and *Th. intermedium* have been conducted, and a series of wheat-*Th. intermedium* partial amphiploids have been developed [16,17]. Recently, Luo et al. [18] developed the wheat-*Th. intermedium* partial amphiploid line 92048, which exhibited resistance to stripe rust races and Fusarium head blight. Utilizing these intermediate materials, dozens of wheat-*Th. intermedium* addition or substitution lines have been developed. Yu et al. [19] bred a set of wheat-*Th. intermedium* addition lines derived from wheat-*Th. intermedium* partial amphiploid TAI7045 and 78784, and 2St and 4St chromosomes exhibited high resistance to powdery mildew. In addition, a novel wheat-*Th. intermedium* T6StS.6J^S^L (6B) disomic substitution line was created for rust resistance breeding [20]. However, addition or substitution lines carrying complete *Th. intermedium* group-6 chromosomes have rarely been reported.

Fluorescence in situ hybridization (FISH) and genomic in situ hybridization (GISH) have been widely applied to distinguish the three *Th. intermedium* subgenomes (J, J^S^ and St) and to identify *Th. intermedium* chromosomes in wheat backgrounds [21]. For example, Yang et al. [22] characterized five *Th. intermedium* 7J^S^ translocation lines by GISH using total genomic DNA from *Th. intermedium* as a probe. Li et al. [23] characterized the wheat-*Th. intermedium* substitution line X479 using the J^S^ chromosome-specific repetitive sequence pDb12H. Non-denaturing FISH (ND-FISH) technology, combined with oligonucleotide (oligo) probes, has been rapidly applied for the detection of alien chromosomes in wheat backgrounds [24]. For instance, a new probe, Oligo-pSt112, derived from high SLAF reads produced distinct signals on terminal regions of 1St#2L [25]. Later, an Oligo-FISH painting system using 45 bp length single-copy sequences as probes was developed for the chromosome identification of Triticeae linkage groups [26].

The objectives of this study were to (1) characterize the genomic constitution of the wheat-*Th. intermedium* double substitution line X482 by Oligo-FISH painting and ND-FISH with multiple probes; (2) perform immuno-FISH of X482 and wheat-*Th. intermedium* partial amphiploid TA8034; and (3) evaluate the powdery mildew resistance of X482-derived lines and physically map the resistance gene(s) on the 6St chromosome. Ultimately, this work aims to provide novel and potentially valuable wheat-*Thinopyrum* germplasm resources for breeding disease-resistant wheat varieties.

## 2. Results

### 2.1. Cytological Characterization of X482 Using ND-FISH and Oligo-FISH Painting

**Figure 1:**
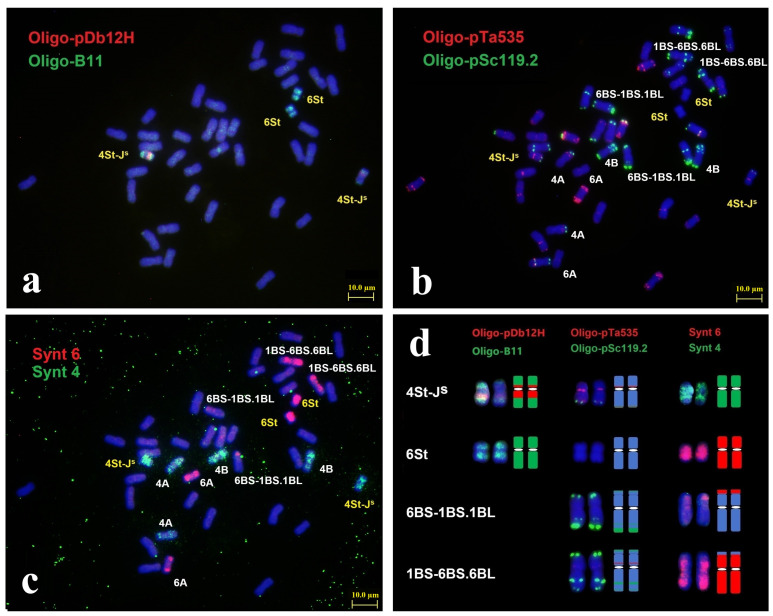
Karyotyping of mitotic metaphase of wheat-*Th. intermedium* substitution line X482 by sequential ND-FISH and Oligo-FISH painting. Sequential ND-FISH by probes: (**a**) Oligo-pDb12H (red) + Oligo-B11 (green), (**b**) Oligo-pTa535 (red) + Oligo-pSc119.2 (green), and (**c**) Oligo-FISH by bulk painting with probes Synt 6 (red) + Synt 4 (green), respectively. The diagrams of 4St-J^S^, 6St, 6BS-1BS.1BL and 1BS-6BS.6BL are shown in (**d**). Bars, 10 μm.

Sequential ND-FISH with probes Oligo-B11, Oligo-pDb12H, Oligo-pSc119.2, and Oligo-pTa535 were used to characterize the chromosome constitution of X482 (Figure 1a,b). Probes Oligo-B11 and Oligo-pDb12H permitted the identification of three subgenomes in *Th. intermedium*. As shown in Figure 1a, X482 contained four *Th. intermedium* chromosomes displaying stronger hybridization signals of Oligo-B11, two of which produced distinct Oligo-pDb12H signals at the long arm and weak Oligo-pDb12H signals at the short arm, which suggested that X482 carried a pair of St chromosomes and a pair of St-J^S^ translocated chromosomes. The probes Oligo-pSc119.2 and Oligo-pTa535 confirmed that X482 had 38 wheat chromosomes, and chromosomes 4D and 6D were substituted by the four *Th. intermedium* chromosomes (Figure 1b). Subsequently, Oligo-FISH painting with probes Synt4 and Synt6 was used to identify the linkage group of alien chromosomes in X482 (Figure 1c). The probe Synt4 produced strong hybrid green signals on the chromosome pairs of 4A and 4B, in addition to the two *Th. intermedium* chromosomes, which were deduced to be 4St-J^S^. Similarly, probe Synt6 generated distinct red signals on the entire lengths of the other two *Th. intermedium* chromosomes, named 6St. Moreover, the unique translocations involving the satellite region of 1B and 6B were detected (Figure 1d), and thus named 1BS-6BS.6BL and 6BS-1BS.1BL. Therefore, X482 was the wheat-*Th. intermedium* double-disomic substitution 4St-J^S^ (4D) + 6St (6D) line, and the ND-FISH hybridization patterns could be used to trace the transmission and variations of chromosomes 4St-J^S^ and 6St in different wheat backgrounds (Figure S1).

### 2.2. Cytological Identification of Wheat-Th. intermedium Amphiploid TA8034

**Figure 2:**
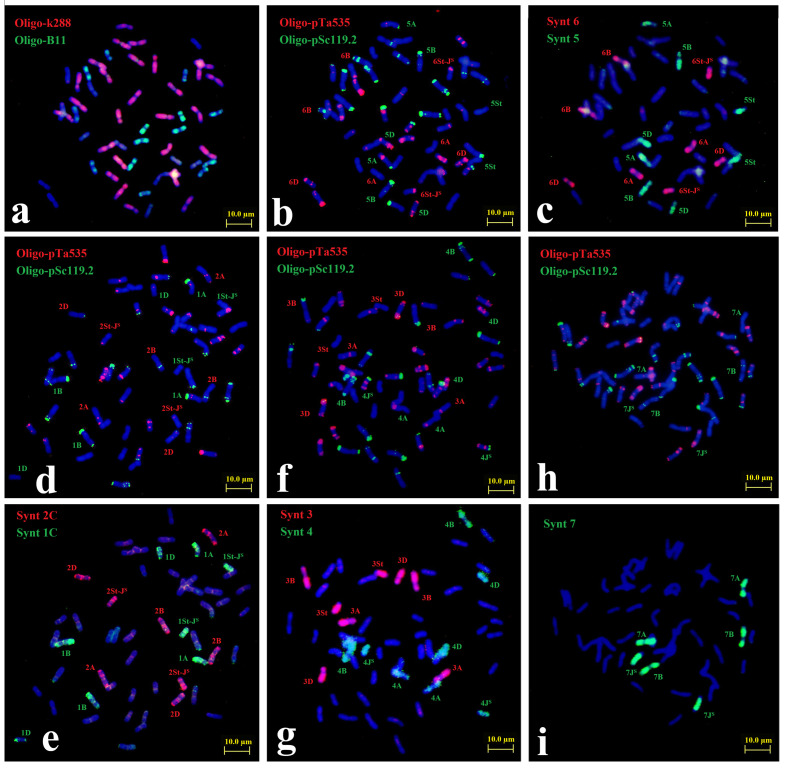
Sequential FISH for TA8034 with multiple probes and oligo-painting. Probes Oligo-B11 + Oligo-k288 were used in combination in (**a**), and probes Oligo-pTa535 + Oligo-pSc119.2 were used in combination in (**b**,**d**,**f**,**h**). Oligo painting probes were Synt6 + Synt5 (**c**), Synt2C + Synt1C (**e**), Synt3 + Synt4 (**g**), and Synt7 (**i**), respectively. Bars, 10 μm.

**Figure 3:**
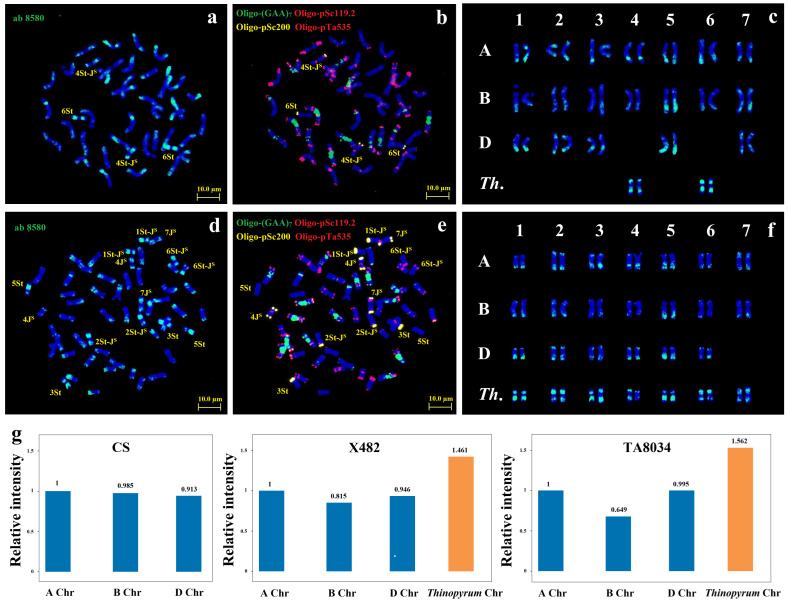
The anti-methylation location and ND-FISH of mitotic metaphase of X482 (**a**–**c**) and wheat-*Th. intermedium* partial amphiploid TA8034 (**d**–**f**). The immunostaining with anti-methylation ab-8580 (**a**,**d**) and sequential ND-FISH by Oligo-pSc119.2 + Oligo-pTa535 + Oligo-pSc200 + Oligo-(GAA)7 (**b**,**e**). Bars, 10 μm. (**c**,**f**) The cut and pasted chromosomes of anti-methylation signals. Scale bars, 10 μm. (**g**) The relative fluorescence intensity of ab-8580 among different subgenomes (compared with A subgenome in each metaphase cell) in Chinese Spring, X482 and TA8034.

As shown in Figure S2, TA8034 contained 40 wheat chromosomes and a total of 14 *Th. intermedium* chromosomes. The ND-FISH patterns of probes Oligo-B11 and Oligo-pDb12H revealed the *Th. intermedium* chromosomes including four St, four J^S^ and six St-J^S^ chromosomes. In addition, the probe Oligo-pSc200 could identify 12 *Th. intermedium* chromosomes in wheat backgrounds. Similarly, ND-FISH with probes Oligo-k288 + Oligo-B11 and Oligo-pTa535 + Oligo-pSc119.2 revealed that the 40 wheat chromosomes each consisted of 14 A, 14 B, and 12 D chromosomes (with an absence of the 7D chromosome) (Figure 2a,b,d,f,h). In combination with bulked pool probes Synt5 + Synt6 (Figure 3c), Synt1C+ Synt2C (Figure 2e), Synt3 + Synt4 (Figure 2g) and Synt7 (Figure 2i), we constructed the karyotype of the 14 *Th. intermedium* chromosomes of TA8034, as linkage groups 1St-J^S^, 2St-J^S^, 3St, 4J^S^, 5St, 6St-J^S^, and 7J^S^, respectively.

### 2.3. Immunostaining Analyses of X482 and Wheat-Th. intermedium Partial Amphiploid TA8034

All chromosomes in line X482 and TA8034 had the CENH3 signals in the centromeric regions, implying that CENH3 protein variants are deposited in the centromeric position in *Th. intermedium* and wheat genomes (Figure 3a,b,f,g). Similarly, three phosphorylation antibodies (AG-3875, AG-3665 and AG-4081) also accumulated in centromeric region of *Th. intermedium* and wheat chromosomes in metaphase (Figure S3c–e,h–j). The methylation locations using antibody ab-8580 were always in paracentric regions of metaphase chromosomes in X482 and TA8034 (Figure 3a,d). Sequential ND-FISH with probes Oligo-(GAA)_7_ + Oligo-pSc119.2 + Oligo-pTa535 + Oligo-pSc200 revealed that the long arm of homologous group 5 chromosomes has the highest methylation modification among wheat chromosomes (Figure 3b,e), and the wheat B subgenome contains relatively fewer methylation sites than the wheat A and D subgenomes (Figure 3g). However, the average relative fluorescence intensity of antibody ab-8580 among A, B, and D subgenomes showed the same level in Chinese Spring (CS) (Figure 3g and Figure S4). The results indicated that *Thinopyrum* chromosomes that introgressed more into the wheat background displayed a larger impact on wheat methylation modification in the B subgenome. Notably, the *Thinopyrum* chromosomes are generally shorter in size; however, they have a significantly higher methylation modification level than those of wheat (Figure 3c,f).

### 2.4. Genetic Analyses and Powdery Mildew Resistance Evaluation of X482-Derived Populations

**Figure 4:**
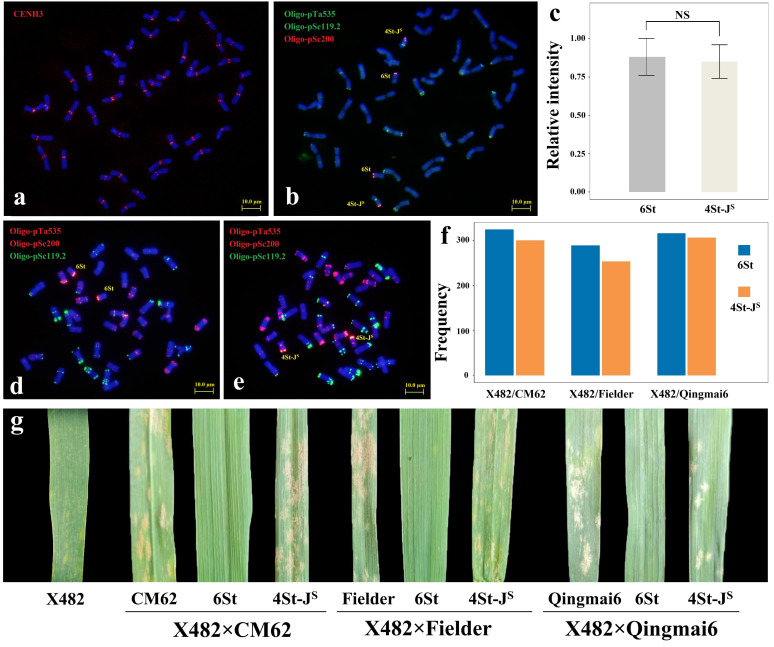
The anti-CENH3 location in X482 (**a**) and sequential ND-FISH by Oligo-pTa535 + Oligo-pSc119.2 + Oligo-pSc200 (**b**). (**c**) The relative fluorescence intensity of CENH3 between chromosome 6St and 4St-J^S^. Bars, 10 μm. “NS” represents “not significant” The karyotype of individuals carrying two 6St (**d**) or 4St-J^S^ (**e**) chromosomes; the probes are Oligo-pTa535 + Oligo-pSc119.2 + Oligo-pSc200. (**f**) The transmission rate of 6St and 4St-J^S^ chromosomes from the hybrids of CM62, Fielder and Qingmai6 with X482. (**g**) Powdery mildew responses of CM62, Fielder, Qingmai6, X482 and their derived lines.

In X482, the average relative fluorescence intensity of CENH3 of chromosomes 6St and 4St-JS was not significant (Figure 4a–c), indicating that the two *Th. intermedium* chromosomes may accumulate enough content of CENH3 protein in the centromere regions during mitosis for reliable chromosome transmission. To transfer and separate the two pairs of *Th. intermedium* chromosomes from X482 to normal wheat, we crossed line X482 with three wheat cultivars, CM62, Fielder and Qingmai6, and then selfed them to produce their F_2_ population. A total of 986 F_2_ plants were produced, including 311, 332 and 343 plants from X482 × CM62, X482 × Fielder and X482 × Qingmai6, respectively. The karyotypes of individual F_2_ plants were screened by ND-FISH using probes Oligo-pSc119.2, Oligo-pTa535 and Oligo-pSc200. Among the 986 F_2_ plants, 43 and 35 had disomic 4St-J^S^ and 6St chromosomes (Figure 4d,e); 96 and 83 had monosomic 4St-J^S^ and 6St chromosomes; and 417 plants contained the mixed karyotypes of 4St-J^S^ and 6St. The remaining 312 plants did not contain *Th. intermedium* chromosomes. The transmission rate of chromosome 6St in three of the crosses was a little higher than that of chromosome 4St-J^S^ (Figure 4f). We also found that three and four lines carried 6StS and 6StL telosomes (Figure S5).

At the adult plant stage, X482 was highly resistant to powdery mildew (IT = 0), whereas CM62, Fielder and Qingmai6 were susceptible to powdery mildew (IT = 4) (Figure 4g). In addition, the evaluation of resistance for the three crosses showed that plants carrying 4St-JS chromosomes were all susceptible to powdery mildew (IT = 3–4). However, plants containing 6St chromosomes were resistant to powdery mildew at the adult plant stage (IT = 0). In the population of X482 × CM62, 6St exhibited a moderate susceptibility to powdery mildew (IT = 3) (Figure S6). Thus, we speculated that *Th. intermedium* chromosome 6St contains adult-stage powdery mildew resistance gene(s) in different wheat backgrounds.

### 2.5. FISH Characterization of Wheat-Th. intermedium 6St Translocation Lines

**Figure 5:**
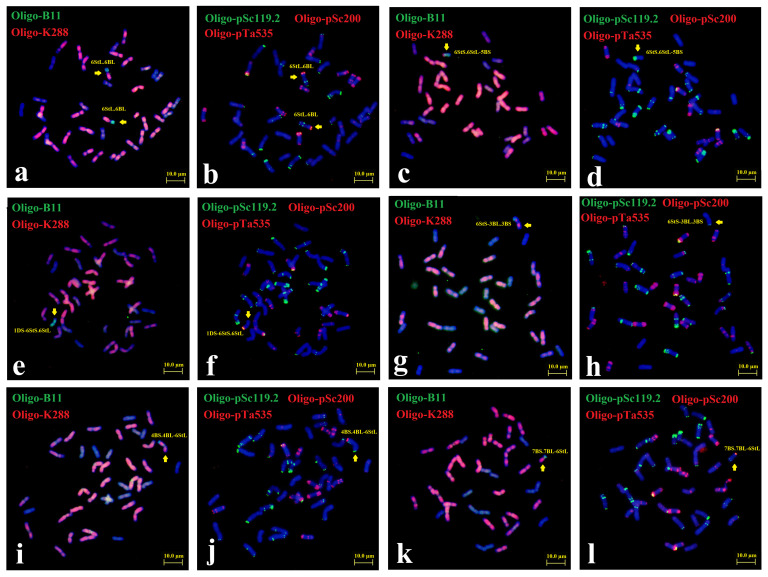
FISH of wheat-*Th. intermedium* 6St translocations 6StL.6BL (**a**,**b**), 6StS.6StL-5BS (**c**,**d**), 1DS.6StL-6StS (**e**,**f**), 6StS-3BL.3BS (**g**,**h**), 4BS.4BL-6StL (**i**,**j**) and 7BS.7BL-6StL (**k**,**l**). The probes were Oligo-k288  +  Oligo-B11 (**a**,**c**,**e**,**g**,**i**,**k**) and Oligo-pTa535 + Oligo-pSc119.2 + Oligo-pSc200 (**b**,**d**,**f**,**h**,**j**,**l**). Bars, 10 µm. Yellow arrows indicated the translocated chromosomes.

We treated the 6St monosomic addition line derived from the progeny of a cross between X482 and CM62 with ^60^Co-γ irradiation. A total of 312 M_1_ plants carrying the monosomic 6St chromosome were selected using the probe Oligo-pSc200. Sequential ND-FISH was performed on 515 self-pollinated M_2_-M_3_ progenies. As a result, seven plants harboring a single aberration type involving chromosome 6St were identified. As shown in Figure 5a,b, we characterized a whole-arm translocation line J45 with 6StL.6BL. In addition, we identified two large-fragment homozygous translocation lines using Oligo-B11 and Oligo-K288. For example, line J66 possessed 41 chromosomes, comprising 40 wheat chromosomes and a translocation involving 6St and an A/B-genome chromosome (Figure 5c). Sequential ND-FISH with Oligo-pTa535, Oligo-pSc119.2, and Oligo-pSc200 revealed a strong green signal in the subterminal region, indicating that a segment of 5BS was fused to the distal region of 6StL, forming the 6StS.6StL-5BS translocation chromosome (Figure 5d). Line J99 carried the homozygous wheat-6St translocation 1DS.6StS-6StL, and ND-FISH localized the breakpoint to the subterminal region of 6StS (Figure 5e,f). Additionally, four small-fragment homozygous translocation lines were developed, including two with long-arm translocation lines, J102 (6StS-3BL.3BS) and J112 (6StS-W), and two with short-arm translocation lines, J12 (4BS.4BL-6StL) and J149 (7BS.7BL-6StL) (Figure 5g–l and Figure S7).

### 2.6. Construction of Physical Maps

**Figure 6:**
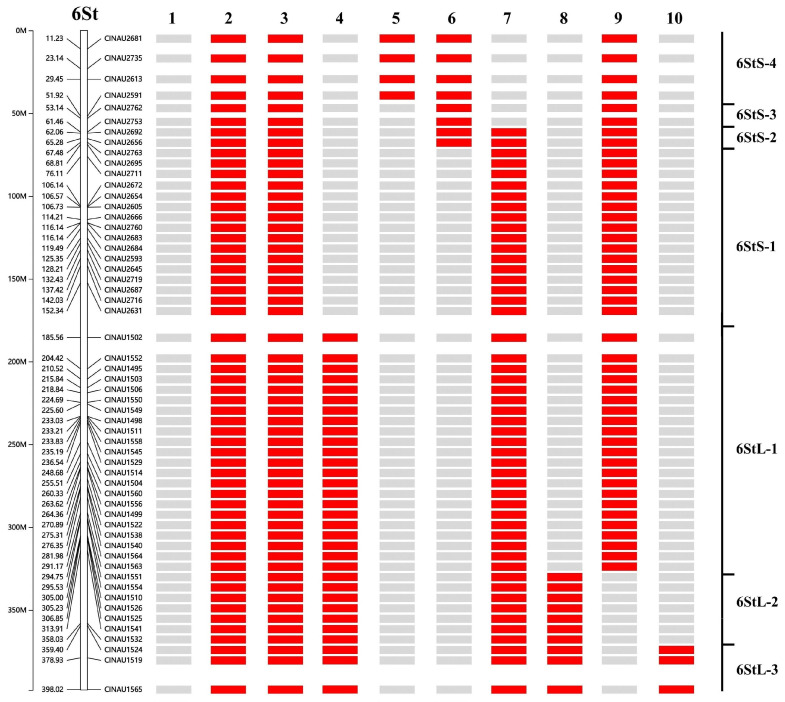
The physical map of chromosome 6St. A total of 56 6St-specific markers were blasted to determine their locations on the genome sequence of 6St from *Th. intermedium*. 1, CM62; 2, X482; 3, 6St carrier; 4, J45; 5, J112; 6, J102; 7, J99; 8, J149; 9, J66; and 10, J12. The diagram on the right shows the different amplification types of seven 6St aberrations. The red box represents amplification, while the grey box represents absence of the 6St-specific bands.

To construct a physical map of the *Th. intermedium* 6St chromosome, we screened 162 intron primers to identify 6St-specific PCR markers. Among these, 56 primer pairs (34.5%) amplified specific bands in materials containing the 6St chromosome and in the parent line X482. Of these markers, 24 were assigned to the short arm 6StS and 32 to the long arm 6StL. We subsequently used 55 of the 6St-specific markers to determine the breakpoint positions in seven wheat-*Th. intermedium* translocation lines. The results showed that the *Th. intermedium* 6St chromosome could be divided into seven bins (Figure 6). Specifically, four, two, two, 16, 22, six, and four markers were assigned to the chromosomal bins 6StS-4, 6StS-3, 6StS-2, 6StS-1, 6StL-1, 6StL-2, and 6StL-3, respectively. Representative markers for different breakpoint types are shown in Figure S8. For example, line J12 only has amplification by primer CINAU1565, which is located within bin 6StL-3, while line J112 is only amplified by primer CINAU2681, located within bin 6StS-4. The constructed physical map was further used to localize the powdery mildew resistance locus on chromosome 6St.

### 2.7. Responses of 6St Translocation Lines to Powdery Mildew

**Figure 7:**
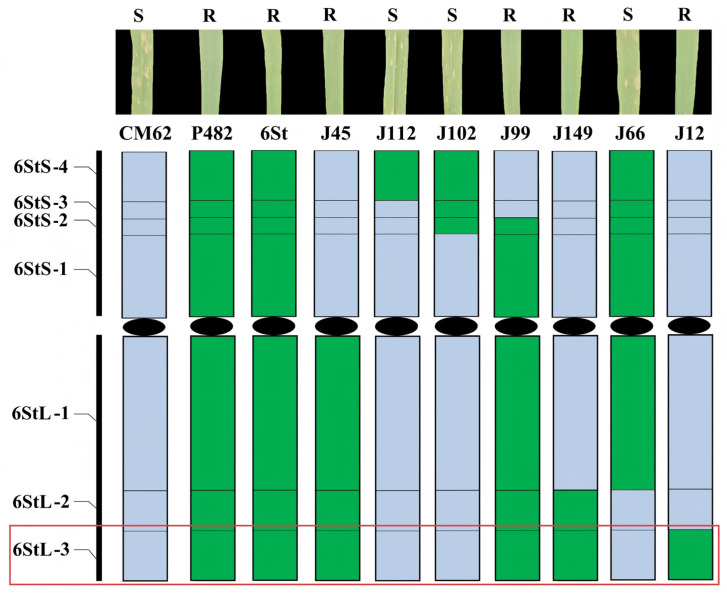
Physical mapping of stripe rust-resistant gene using seven 6St aberration lines. Green colors indicate chromosome segments of 6St; blue represents wheat chromosome fragments. The red box indicates the physical region of the *Pm* locus.

We subsequently evaluated the adult-stage reactions of the seven wheat-*Th. intermedium* 6St aberration lines. Two lines (J102 and J66) were all powdery mildew susceptible, and four lines (J45, J99, J149 and J12) containing bin 6StL-3 were all powdery mildew resistant like the parental line X482 and 6St carrier. According to the physical positions of these specific markers, the *Pm* locus in bin 6StL-3 was located on the FL0.82–1.00 of 6StL and corresponded to the terminal 358.03–398.21 Mb in the reference *Th. intermedium* genome sequence (Figure 7). According to the gene annotation, a total of 628 genes (*Thin6S508900.1*-*Thin6S571700.1*) have been identified in this region. Among them, twelve genes were annotated as LRR disease resistance protein or receptor-like kinase protein (Table S1).

### 2.8. Evaluation of Major Agronomic Traits of X482 and Its Derived Lines

**Figure 8:**
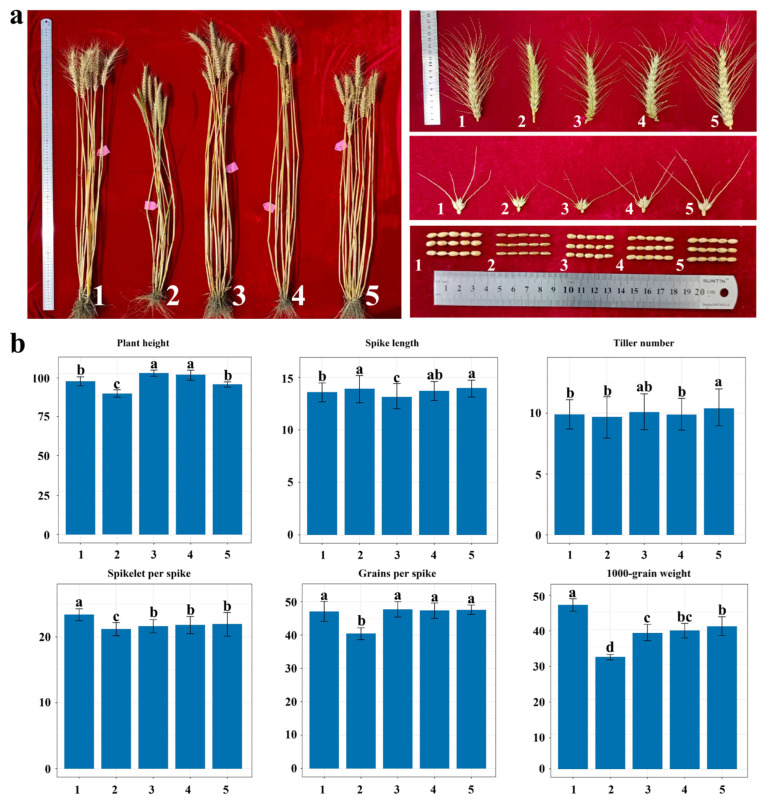
Plant morphology (**a**) and statistical analysis of six agronomic traits (**b**). 1, CM62; 2, X482; 3, 6StS carrier; 4, 6StL carrier; and 5, J45. “a, b, c and d” indicate the statistical significance of differences evaluated by one-way ANOVA. Significance is noted at the *p*  <  0.05 level. Different letters denote significant differences, while the same letter indicates no significant difference.

The yield performance of the two parental lines, X482 and CM62, as well as the 6StS addition lines, 6StL addition lines, and the powdery mildew-resistant translocation line J45, were evaluated during the 2024–2025 growing season. The PH, grains per spike, SS and TGW of X482 were significantly lower than those of CM62. The 6StS and 6StL addition lines exhibited no significant differences in PH, TN, SS, grains per spike and TGW, but displayed an increase in plant height compared to CM62. In contrast, J45 showed lower plant height and higher tiller number in comparison to CM62 (Figure 8). Accordingly, the derivatives carrying the 6St chromosome segment conferring powdery mildew resistance had no adverse effect on wheat yield-related traits, indicating their potential utility in wheat breeding programs.

## 3. Discussion

The narrow genetic base of modern bread wheat (*Triticum aestivum* L.) cultivars poses a significant challenge to sustainable yield improvement. Introgression of genetic diversity from wild relatives offers a promising strategy to enhance wheat breeding efforts for global food security [27]. *Thinopyrum intermedium* is a valuable perennial wild relative of hexaploid wheat, harboring multiple disease resistance genes that can be effectively transferred into cultivated wheat. In this study, we precisely characterized the wheat-*Th. intermedium* derivative line X482, which exhibits high-level resistance to powdery mildew in the field. Cytogenetic analyses, including ND-FISH and Oligo-FISH painting, confirmed that X482 is a stable double-disomic substitution line with the constitution 4St-J^S^ (4D) + 6St (6D), and additionally carries two reciprocal translocations involving the satellite regions of chromosomes 1B and 6B. Subsequent genetic analyses in three hybrid populations enabled the characterization of multiple *Th. intermedium* 6St translocation lines. These findings confirmed X482 as a novel wheat-*Th. intermedium* germplasm resource and demonstrated that ND-FISH combined with Oligo-FISH painting is highly effective for the precise identification of alien chromosomes in a wheat background. Furthermore, the 6St translocation lines developed in this study provide valuable materials for the rapid utilization of elite traits from chromosome 6St in common wheat breeding.

Chromosomal rearrangements (CRs) play a critical role in maintaining genome stability and facilitating environmental adaptation during speciation [28]. In Triticeae species, extensive structural reorganization often arises from wide hybridization events and can be perpetuated in subsequent generations. Among various CRs, Robertsonian translocations are the most prevalent, exemplified by the widely deployed 1RS.1BL translocation in wheat breeding, wherein the short arm of wheat chromosome 1B is replaced by that of rye chromosome 1R [29]. In addition to centromeric regions, secondary constrictions may also serve as fragile sites prone to breakage [30]. However, reports of CRs involving primary constrictions remain relatively scarce. Guo et al. [31] first characterized the stripe rust-resistant line Zhongke 15, which harbors a *Th. intermedium* insertion within the satellite region of chromosome 6B and exhibits favorable agronomic traits. Similarly, Sestili et al. [32] identified a disomic addition line derived from triticale × wheat crosses that carried a centromeric breakage of chromosome 1R, with its long arm fused to the satellite of wheat chromosome 6B; both the deletion and translocation chromosomes exhibited pTa71 signals. In the present study, we identified translocations involving the rearranged satellite regions of wheat chromosomes 1B and 6B using Oligo-FISH painting. These translocations displayed normal Oligo-pTa71 and Oligo-(GAA)_7_ signals and could not be distinguished by other probes such as Oligo-pSc119.2 (Figure S9). Whether such translocations arise from genomic shock induced by wheat-*Th. intermedium* hybridization or pre-exist in the wheat background, and whether they affect agronomic performance or gene expression, warrant further investigation.

Histone modifications, including acetylation, methylation, phosphorylation, and ubiquitination, play pivotal roles in modulating chromatin structure and facilitating DNA accessibility for the transcriptional machinery. Among these, phosphorylation of histone H3 at serine 10 (H3S10ph) is closely associated with the initiation and maintenance of mitotic chromosome condensation [33]. Using immunofluorescence labeling with an H3S10ph-specific antibody, Yang et al. [34] observed predominant signal accumulation in pericentromeric regions during metaphase in wheat mitotic divisions, a pattern similarly reported in *Secale cereale* and *Hordeum vulgare* [35]. In contrast, Song et al. [36] found that H3S10ph was distributed across entire chromosome arms throughout all mitotic stages in frozen wheat root tip cells, suggesting that spatial dynamics may vary with tissue treatment or experimental conditions. In our study, three antibodies targeting phosphorylated histones H3 and H2A all showed preferential deposition at centromeric regions on metaphase chromosomes in line X482 and the wheat-*Th. intermedium* partial amphiploid TA8034, indicating that both wheat and *Th. intermedium* share similar patterns of histone phosphorylation distribution. Moreover, the introduction of *Th. intermedium* chromosomes into the wheat background did not result in detectable hyperphosphorylation or dephosphorylation of histones on metaphase chromosomes. Notably, the DNA methylation level of *Th. intermedium* chromosomes in X482 and TA8034 was higher than that of common wheat chromosomes, consistent with previous reports suggesting that introgressed alien chromatin is often associated with suppressed gene expression [37]. Interestingly, as the number of introduced alien chromosomes increased, the overall DNA methylation level of the wheat B subgenome further decreased. Collectively, these observations provide a foundation for further exploration of the epigenetic consequences of alien introgression in wheat.

Powdery mildew is one of the most destructive foliar diseases in wheat and typically reduces wheat yield by 10–15%, and up to 50% in more severe instances. Up until now, 71 powdery mildew resistance genes have been formally designated, and three of them are derived from the *Thinopyrum* genus. For instance, *Pm40* on wheat chromosome 7BS was found in the wheat-*Th. intermedium* introgression line GRY19 [13]; *Pm43* was characterized in wheat-*Th. intermedium* introgression line CH5052 with the dominant inheritance on chromosome 2DL [14]; and line CH7086 carrying *Pm51* was selected from the progenies of Xiaoyan 7430 [38]. In addition, a series of wheat-*Th. intermedium* introgression lines carrying resistance to powdery mildew have been given temporary names, such as *PmL692* and *PmWE99* [39]. Zhan et al. [40] developed the compensating Robertsonian translocation line CH13–21 (T6BS.6Ai#1L) conferring resistance to powdery mildew and stripe rust. However, the precise physical locations of these resistance genes within the alien genome often remain unclear. In the present study, we located the *Pm* genes on 6StL, and chromosome arm 6StS was susceptible to powdery mildew. In the present study, we selected seven 6St translocation lines from the M_2_-M_3_ generations and mapped a powdery mildew resistance gene to the FL 0.82–1 region on 6StL, corresponding to the physical intervals of 622.21–630.85 Mb on chromosome 6A, 741.37–754.87 Mb on chromosome 6B, and 497.98–507.59 Mb on chromosome 6D in the reference Chinese Spring T2T assembly [41]. *Pm20* and *Pm27* were characterized on the long arm of 6R and 6G from Rye and *Triticum timopheevii*, respectively [5]. Moreover, *Pm54* derived from the soft red winter wheat line 26R61 was mapped to the subterminal region of chromosome 6BL, with the closely linked marker Xbarc134 at 729.76 Mb on 6BL [42]. Similarly, Li et al. [43] cloned *Pm69* in the telomeric region of chromosome 6BL in *Triticum dicoccoides*, linked with molecular markers *uhw367* and *uhwk389*, corresponding to a physical interval of 748.68–748.69 Mb on Chinese Spring 6BS. Recently, two wheat–rye translocation lines, 6BS.6BL-6RL^Ku^ and 6BS.6RL^Ar^, were developed, with both carrying powdery mildew resistance genes mapped to approximately 100 Mb of the distal segment of 6RL [44,45]. These findings suggest that the distal regions of homologous group 6 chromosomes harbor numerous resistance genes with potential for further exploration and utilization. Meanwhile, whether these resistance genes are allelic remains to be determined through further fine mapping of the resistance regions.

## 4. Materials and Methods

### 4.1. Plant Materials

The wheat-*Th. intermedium* line X482 (2n = 6x = 42) [46], wheat cultivar Chuanmai62 (CM62), Qingmai6 and Fielder, and wheat-*Th. intermedium* partial amphiploid TA8034 (2n = 6x = 54) are maintained in our laboratory at the University of Electronic Science and Technology of China. *Th. intermedium* PI440043 (StStJ^S^J^S^JJ, 2n = 6x = 42) was obtained from the National Small Grains Collection at Aberdeen, ID, USA.

### 4.2. ND-FISH and Oligo-FISH Painting

The root-tip metaphase chromosomes of all germinated seeds were prepared according to the procedure described by Han et al. [47]. The synthetic oligonucleotides Oligo-pSc119.2, Oligo-pTa535, Oligo-B11, Oligo-K288, Oligo-pDb12H and Oligo-pSc200 were used for ND-FISH analysis and their sequences are shown in Table 1. All oligonucleotide probes were either 5′ end-labeled with 6-carboxyfluorescein (6-FAM) for green signals or 6-carboxytetramethylrhodamine (Tamra) for red signals. After the ND-FISH analysis of metaphase chromosomes, the Oligo-FISH painting technique was performed following the description by Li and Yang [48]. The pictures of FISH results under Olympus BX-53 microscope were taken by a DP-70 CCD camera (Japan).

### 4.3. Chromosomal Immunolocalization

The rabbit polyclonal antibody anti-CENH3 was purified by affinity chromatography [54] and was produced by GL Biochem Ltd. (Shanghai, China). In addition, the anti-phosphorylation AG3875 (mouse monoclonal antibody) and AG3665 (rabbit polyclonal antibody) from Beyotime Biotechnology, Shanghai, China, and anti-methylation ab8580 (rabbit polyclonal antibody) from Abcam, USA, were used to detect anti-CENH3 hybridization sites. Immunolocalization on mitotic metaphase chromosomes was performed as described previously [55]. The locations of anti-CENH3 hybridizations on chromosomes utilized CENH3 (1:300)/phosphorylation (1:500)/methylation (1:500) and goat anti-rabbit Texas green (1:1000)/goat anti-mouse Texas red (1:1000) (Sigma-Aldrich, St. Louis, MO, USA). The images were collected with the BX53 Motorized System Microscope (Olympus, Japan) and processed using Adobe Photoshop CS 4.0 (Adobe, USA).

### 4.4. Molecular Marker Analysis

DNA of all materials used in this study was extracted from young leaves using the SDS protocol [8]. CINAU markers of linkage group 6 based on intron length polymorphisms were obtained from Zhang et al. [56] and Zhang et al. [57]. The specific 6St markers were searched for physical chromosomal locations using the database *Thinopyrun intermedium* genome V.2.1 (https://phytozome-next.jgi.doe.gov/info/Tintermedium_v2_1, accessed on 21 January 2021). The amplification program involved 4 min at 94 °C, 35 cycles of 45 s at 94 °C, 45 s at 58 °C, 1 min at 72 °C and a final extension at 72 °C for 10 min. PCR amplification products were analyzed by 1% agarose gel electrophoresis and 8% PAGE gel electrophoresis, as described by Jiang et al. [58].

### 4.5. Powdery Mildew Infection Scoring

The reactions of the parent wheat CM62, Fielder, Qingmai6, the double 4St-J^S^ (4D) + 6St (6D) substitution line X482, and their derived progenies to powdery mildew were assessed at adult-plant stages during the 2024–2025 season. The inoculation of local mixed Bgt isolates was carried out at the four-leaf stage in a greenhouse at 23 °C and the response was recorded at 20 days post-inoculation. Infection types were scored according to the system described by Bariana and McIntosh [59].

### 4.6. Evaluation of Agronomic Traits

During the 2024–2025 growing seasons, the translocation line J45 (6StL.6BL), the 6StS and 6StL addition lines and their parent lines X482 and CM62 were grown at the Xindu Experimental Station, Chengdu, China. Ten seeds were planted in a row of 1.5 m in length with an inter-row spacing of 0.25 m. Six agronomic traits were evaluated: plant height (PH), tiller number (TN), spike length (SL), spikelets per spike (SS), grains per spike, and thousand-grain weight (TGW). The mean value and standard deviation were calculated by SPSS Statistics19.0 software. For multiple comparisons among groups, one-way ANOVA followed by Tukey’s HSD post hoc test was applied.

## Figures and Tables

**Table 1:** The sequences of oligo probes for *Thinopyrum* chromosome identification by ND-FISH.

Oligo Probes	Sequences	Reference
Oligo-pTa535	AAAAACTTGACGCACGTCACGTACAAATTGGACAAACTCTTTCGGAGTATCAGGGTTTC	[49]
Oligo-pSc119.2	CCGTTTTGTGGACTATTACTCACCGCTTTGGGGTCCCATAGCTAT	[49]
Oligo-K288	TATTGATGATATGGGTAGTACAAGAGGAGATCTACCACGAGATCAGAGAGGCTAAACCC	[50]
Oligo-pSc200	CTCACTTGCTTTGAGAGTCTCGATCAATTCGGACTCTAGGTTGATTTTTGTATTTTCT	[51]
Oligo-B11	TCCGCTCACCTTGATGACAACATCAGGTGGAATTCCGTTCGAGGG	[52]
Oligo-pDb12H	TCAGAATTTTTAGGATAGCAGAAGTATTCGAAATACCCAGATTGCTACAG	[53]
Oligo-pTA71	GGGCAAAACCACGTACGTGGCACACGCCGCGTA	[53]
Oligo-(GAA)_7_	GAAGAAGAAGAAGAAGAAGAA	[53]

## Data Availability

Data are contained within the article.

## Supplementary Materials

The following supporting information can be downloaded at https://www.mdpi.com/article/10.3390/plants15091308/s1: Figure S1: Karyotypes of X482 with homologous and subgenome assignment using Oligo-pSc119.2 + Oligo-pTa535. Figure S2: Sequential FISH for TA8034 with multiple probes. Probes Oligo-B11 + Oligo-pDb12H were used in combination in (a), probes Oligo-pTa535 + Oligo-pSc119.2 were used in combination in (b), and probe Oligo-pSc200 was used in (c), respectively. Bars, 10 μm. Figure S3: The anti-CENH3 (a,f), anti-phosphorylation AG-3875/AG-3665/AG-4081 (c–e,h–j) and sequential ND-FISH with probes Oligo-pSc119.2 + Oligo-pTa535 + OligopSc200 (b) and Oligo-pSc119.2 + Oligo-pTa535 + OligopSc200 + Oligo-(GAA)7 (g) in X482 (a–e) and wheat-*Th. intermedium* partial amphiploid TA8034 (f–j). Bars, 10 μm. Figure S4: The anti-methylation location and ND-FISH of mitotic metaphase of Chinese Spring. Bars, 10 μm. Figure S5: Sequential FISH for 6StS telosomes (a,b) and 6StL telosomes (c,d). Probe were: Oligo-B11 + Oligo-K288 (a,c) and Oligo-pSc119.2 + Oligo-pTa535 +Oligo-pSc200 (b,d). Bars, 10 µm. Figure S6: Powdery mildew responses of CM62, X482 and their derived lines at seeding stage. Figure S7: Karyotype of 6StS-W translocation with probes Oligo-k288  +  Oligo-B11 (a) and Oligo-pTa535  +  Oligo-pSc119.2 + Oligo-pSc200 (b). Bars, 10 µm. Figure S8: PCR amplification results of representative 6St-specifc markers in the seven translocation lines. 1, CM62; 2, X482; 3, 6St carrier; 4, J45; 5, J112; 6, J102; 7, J99; 8, J149; 9, J66; and 10, J12. Arrows point to chromosome 6St-specifc bands. Figure S9: Sequential FISH for X482 with probe Oligo-pSc119.2 + Oligo-pTa535 (a), Oligo-pTa71 (b) and Oligo-(GAA)7. Bars, 10 µm. Table S1: LRR resistance-like genes and receptor-like kinase genes information.
