# Supplementary material for: Physical Mapping of a Powdery Mildew Resistance Gene in Chromosome 6St from Wheat-Thinopyrum intermedium Introgression Lines
Source: Plants (Basel). 2026 Apr 24;15(9):1308. doi: 10.3390/plants15091308 (PMC13164910; doi:10.3390/plants15091308)
Supplement: Supplementary file 1 [file plants-15-01308-s001.zip › plants-4258381-supplementary.pdf]

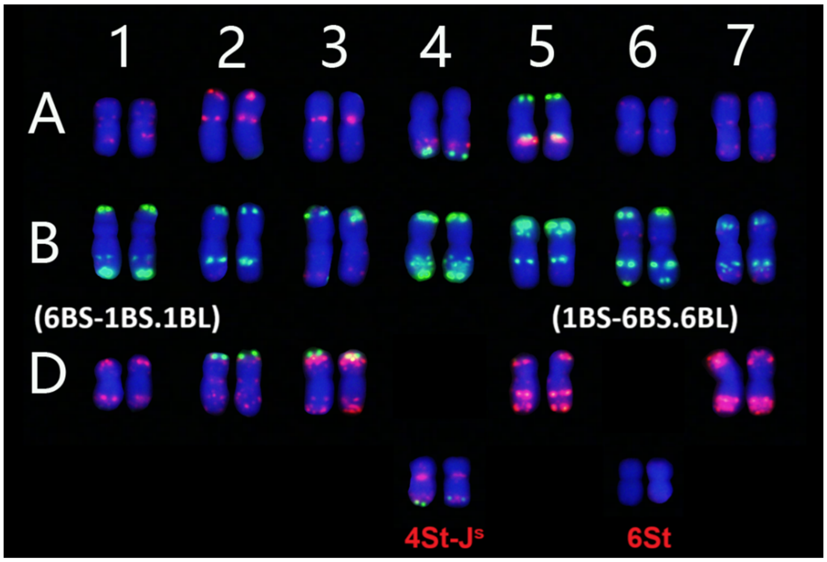

Figure S1. Karyotypes of X482 with homoeologous and subgenome assignment using Oligo-pSc119.2 + Oligo-pTa535.

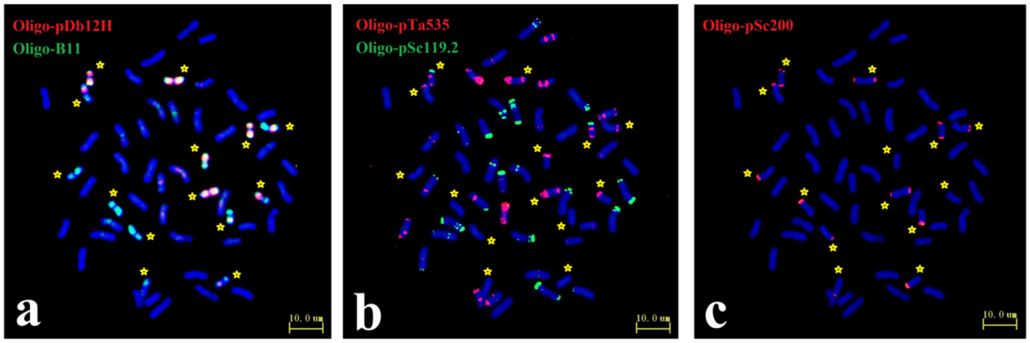

Figure S2. Sequential FISH for TA8034 with multiple probes. Probes Oligo-B11 + Oligo-pDb12H were used in combination in (a), probes Oligo-pTa535 + Oligo-pSc119.2 were used in combination in (b), and probe Oligo-pSc200 was used in (c), respectively. Bars, 10 µm.

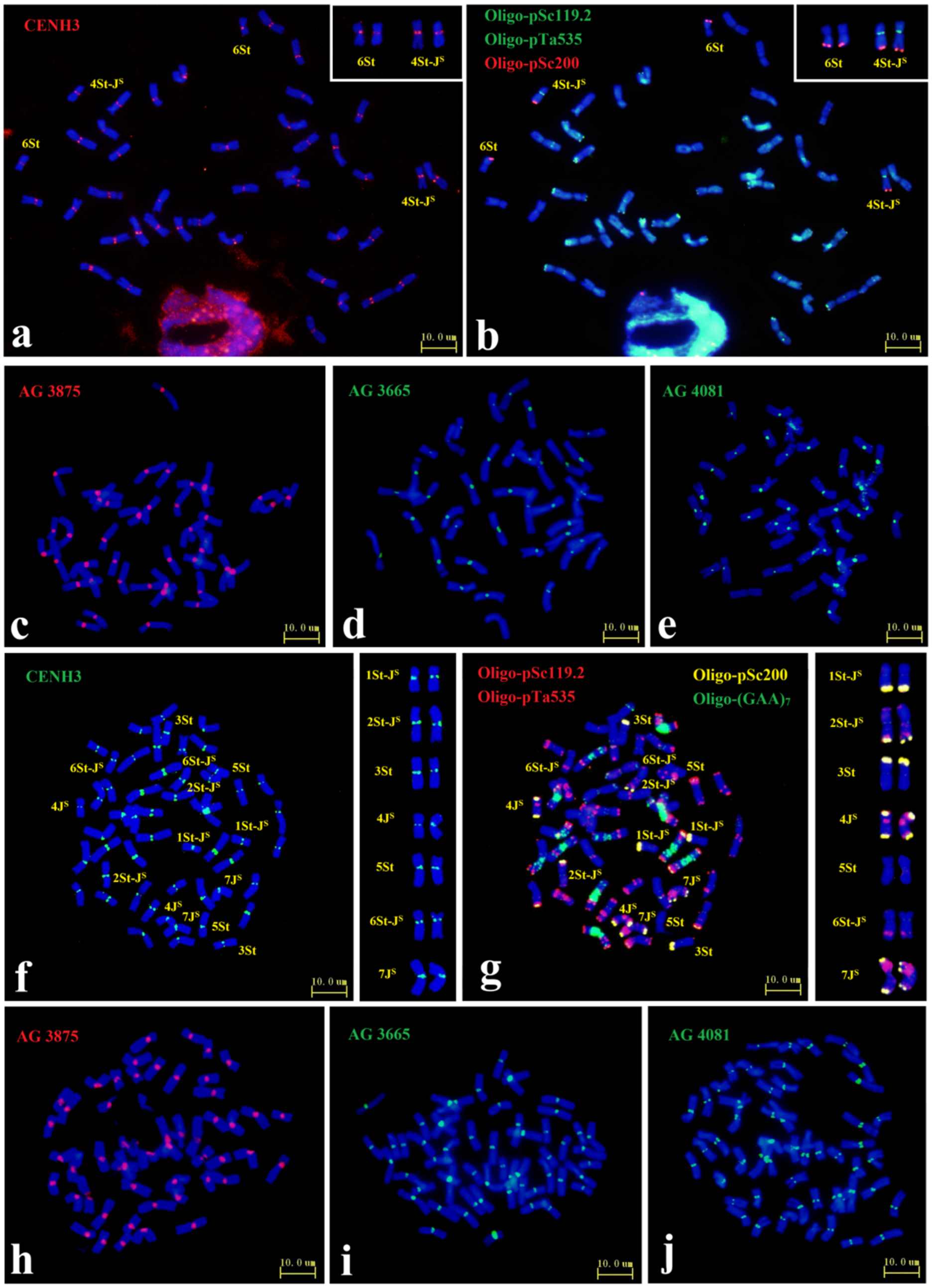

Figure S3. The anti-CENH3 (a, f), anti-phosphorylation AG-3875/AG-3665/AG-4081 (c-e, h-j) and sequential ND-FISH with probes Oligo-pSc119.2 + Oligo-pTa535 + OligopSc200 (b), Oligo-pSc119.2 + Oligo-pTa535 + OligopSc200 + Oligo-(GAA)$_7$ (g) in X482 (a-e) and wheat-*Th. intermedium* partial amphiploid TA8034 (f-j). Bars, 10 μm.

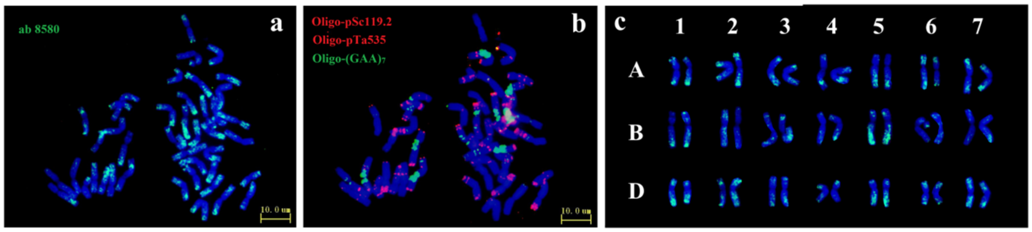

Figure S4. The anti-methylation location and ND-FISH of mitotic metaphase of CS. Bars, 10 μm.

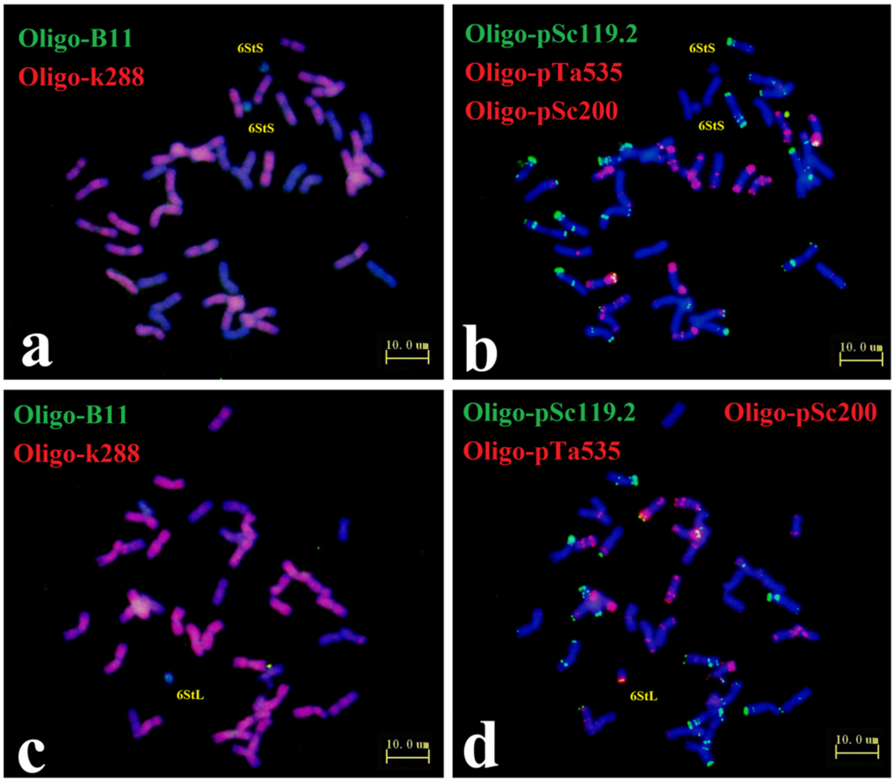

Figure S5. Sequential FISH for 6StS telosomes (a-b) and 6StL telosomes (c-d). Probe were: Oligo-B11 + Oligo-K288 (a, c) Oligo-pSc119.2 + Oligo-pTa535 +Oligo-pSc200 (b, d). Bars, 10 μm.

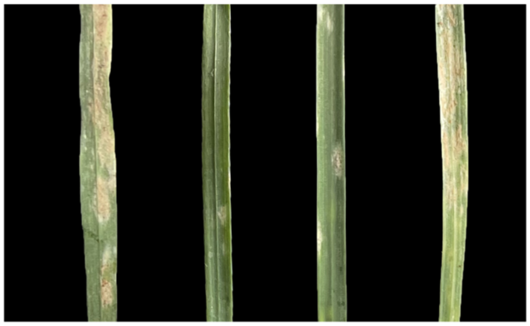

Figure S6. Powdery mildew responses of CM62, X482 and their derived lines at seeding stage.

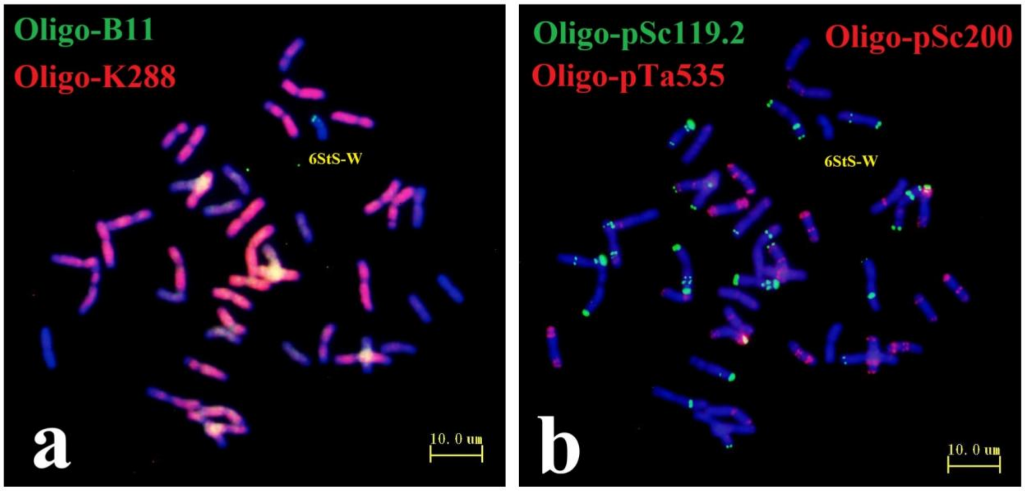

Figure S7. Karyotype of 6StS-W translocation with probes Oligo-k288 + Oligo-B11 (a), Oligo-pTa535 + Oligo-pSc119.2 + Oligo-pSc200 (b). Bars, 10 µm.

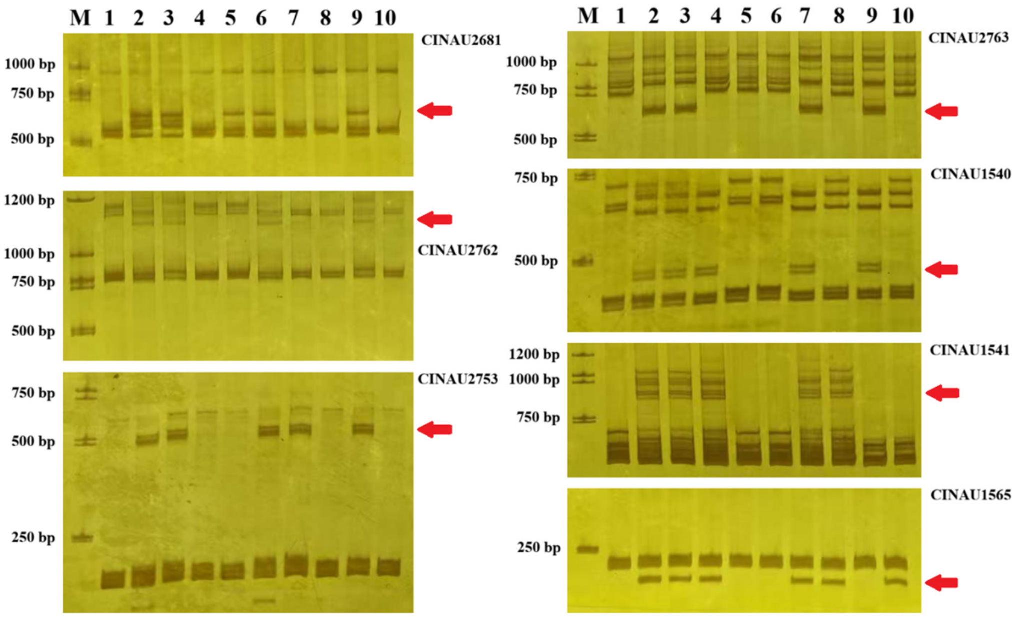

Figure S8. PCR amplification results of representative 6St-specifc markers in the seven translocation lines. 1, CM62; 2, X482; 3, 6St carrier; 4, J45; 5, J112; 6, J102; 7, J99; 8, J149; 9, J66; 10, J12. Arrows point to chromosome 6St-specifc bands.

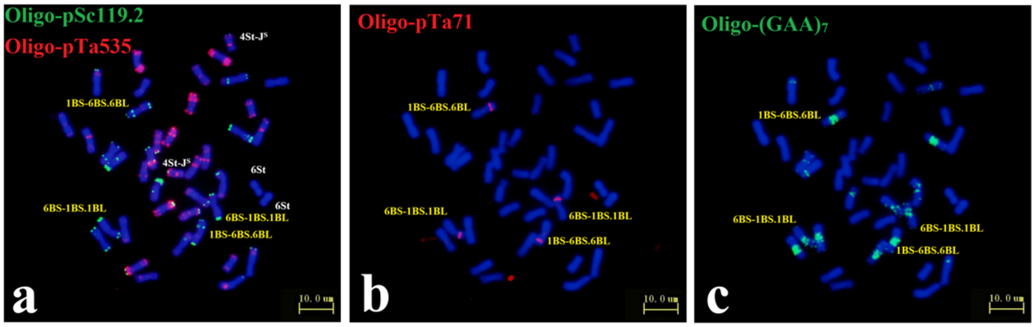

Figure S9. Sequential FISH for X482 with probe Oligo-pSc119.2 + Oligo-pTa535 (a), Oligo-pTa71 (b) and Oligo-$(GAA)_7$. Bars, 10 µm.

Table S1 LRR resistance-like genes and receptor-like kinase genes information

Gene ID	Chromosome	Start position	Functional annotation
Thin6S546000.1	6St	385763817	LRR disease resistance protein
Thin6S547400.1	6St	386303254	LRR disease resistance protein
Thin6S558800.1	6St	392253830	LRR disease resistance protein
Thin6S559000.1	6St	392351063	LRR disease resistance protein
Thin6S559800.1	6St	392890131	LRR disease resistance protein
Thin6S559900.1	6St	393012367	LRR disease resistance protein
Thin6S560000.1	6St	393263486	LRR disease resistance protein
Thin6S560100.1	6St	393343679	LRR disease resistance protein
Thin6S560800.1	6St	393725484	LRR disease resistance protein
Thin6S568900.1	6St	396942039	LRR disease resistance protein
Thin6S569100.1	6St	397086981	LRR disease resistance protein
Thin6S569200.1	6St	397112782	LRR disease resistance protein
